# Contribution of base diet, voluntary fortified foods and supplements to micronutrient intakes in the UK

**DOI:** 10.1017/jns.2022.47

**Published:** 2022-06-23

**Authors:** Julia K. Bird, Rebecca Barron, Sandrine Pigat, Maaike J. Bruins

**Affiliations:** 1 Bird Scientific Writing, Wassenaar, the Netherlands; 2 Creme Global, Dublin, Ireland; 3 DSM Nutritional Products, Kaiseraugst, Switzerland

**Keywords:** Dietary survey UK, Fortification, Micronutrients, Inadequacies, Supplements

## Abstract

The objective of the present study was to evaluate the contribution of voluntary fortified foods and supplements to reducing micronutrient shortfalls in the UK population. A secondary analysis of the UK National Diet and Nutrition Survey was conducted (2012/13–2013/14, *N* 2546, 1·5–95 years). Micronutrient intakes were derived from food consumption intake data and food composition data and calculated as the proportion below or above the Dietary Reference Values for males and females of different age groups, for those on a base diet only, users of fortified foods but no supplements and users of fortified foods and supplements. Of the population consuming a base diet only, 21–45 % and 5–29 % fell below the Estimated Average Requirement (EAR) for minerals and vitamins, respectively. About 3–13 % fewer consumers of fortified foods fell below the EAR for vitamins and minerals. Supplements barely reduced the prevalence of intakes below the EAR. Among supplement non-users and users, 99 and 96 % failed to meet the reference intakes for vitamin D. More women than men were at risk of inadequacies of micronutrient intakes. The prevalence of inadequacies declined with increasing age. Voluntary fortified foods but not supplements made a meaningful contribution to intakes of vitamin and minerals, without risk of unacceptably high intakes. These insights may help the UK to define approaches to address micronutrients of concern in vulnerable groups.

## Introduction

The National Diet and Nutrition Survey (NDNS) conducted over the past decade shows that the UK population adherence to the dietary recommendations is low^(1–3)^. Intakes of most vitamins and minerals declined over the 9-year period, particularly folate and vitamin A^(3)^. There was little change over 9 years in riboflavin, vitamins B12, C and D, or iron status^(3)^. Analyses using the NDNS have revealed that a high proportion of UK residents, particularly women, had very low intakes for vitamin D, selenium, potassium, iron, riboflavin, magnesium and iodine^(3,4)^.

Different approaches exist to improve vitamin and mineral intakes: improved nutrition education, addition of vitamins and minerals to fortified foods, and/or supplements can help some people meet their nutritional needs. Food fortification, if well-planned, can be effective, safe, and can have a substantial public health impact^(5)^. While recommendations to take vitamin supplements may target those most at risk of inadequate nutrient intakes, uptake can be low^(6)^.

Relatively little is known about the contribution of voluntary fortification and food supplements in the UK to meeting the intake requirements of vitamins and minerals. Therefore, the aim of the present study was to quantify the intakes of micronutrients from the base diet, voluntarily fortified foods and food supplements, and determine the proportion of the UK population outside the UK's Dietary Reference Values (DRVs).

## Methods

### Dataset

The NDNS survey years 5 and 6 (2012/13–2013/14), and the Diet and Nutrition Survey, was used. All NDNS was conducted according to the Declaration of Helsinki and obtained full ethical approval from the necessary committee (Cambridge South National Research Ethics Service (NRES) Committee, Reference 13/EE/0016)^(7)^. Written informed consent was obtained from all subjects. Methods of the NDNS dietary data collection have been described in detail^(7)^. In brief, a total of 2546 participants aged 1·5–95 years were given a repeat 24-hour recall and 4-day estimated (unweighted) food diary for which the start date was randomly assigned, and the time period included weekend days. No data were collected from pregnant or lactating women, or people living in institutions. The dataset demographics have been described previously in detail in a report from Public Health England and the Food Standards Agency^(2)^.

### Dietary intake calculations

The NDNS adult food diary provided photographs of 15 commonly consumed items in small, medium and large portion sizes which participants could use for identical or similar foods. The NDNS child and toddler diaries did not include photographs of foods, so all portion sizes were recorded in household measures or weights or volume from packaging. After each day, participants record if their intake was typical for that day and details of any food supplements taken. Dietary data were processed by trained coders and editors. Food intakes were submitted into a modified version of HNR's dietary assessment system DINO (Diet In Nutrients Out), a Microsoft Access-based all-in-one dietary recording and analysis system. Food consumption data were converted to nutrient intakes using the food composition data of the Department of Health's NDNS Nutrient Databank, which was incorporated into the DINO system^(8)^. Weights for unprocessed foods were published in the Food Standards Agency Food Portion Sizes book, whereas weights for manufactured foods were provided in the Food Standards Agency Food Portion Sizes book. Dietary supplement use was collected as part of the 4-day food diary. Further methodological details on the NDNS dietary data collection are reported in Section 7 of the main report^(9)^ and Appendix A of the extra NDNS material^(10)^.

### Determination of micronutrient intakes from the base diet, fortification and supplements

As per previous publications examining fortified foods in the UK, fortification of flour (with iron, thiamine, calcium and niacin) to restore levels to their original, pre-milled levels were not considered as fortified foods^(11)^. Macronutrients added to foods and supplements contribute little to intakes and were also outside scope of this analysis. The food composition data of the Department of Health's NDNS Nutrient Databank includes information of different types of branded voluntary fortified food brands and a fortification description (e.g., added vitamins, fortified). Fortified foods were identified through either fortification descriptors in the name or through brand name. If the micronutrients a food was fortified with could not be identified, the food was assigned to a general fortified food group, similar to previous published methodologies^(11)^. If a product could not be identified as fortified through the above, then it was assumed to not be fortified. Foods were then grouped into the following based on the micronutrients they were fortified with calcium, folic acid, iron, niacin, magnesium, vitamin A, vitamin B1, vitamin B5, vitamin B6, vitamin B12, vitamin C, vitamin D, vitamin E, undefined and all fortified foods. Undefined fortified foods include foods where ‘fortified’ was in the description, however, the fortified micronutrient(s) could not be determined. A description of foods in each fortified food group is available in Supplementary Table S1. It was not possible within this survey to separate micronutrients in fortified foods into their innate and fortified components, therefore, the contribution represents the total micronutrient content of the food. Intakes for calcium, folate, iodine, iron, magnesium, niacin, pantothenic acid, potassium, riboflavin, selenium, thiamine, vitamin A, vitamin B12, vitamin B6, vitamin C, vitamin D, vitamin E and zinc were estimated using software designed and validated for modelling dietary exposure to food and chemicals^(12)^. Dietary supplements included any multivitamin-mineral or single micronutrient preparation taken. Mean intakes of vitamins and minerals were calculated for the following age and gender categories: age 1–3, 4–6 and 7–10 years with genders combined, and age 11–14, 15–18, 19–50 and 51 years and over for males and females separately. Participants aged 1–9, 10–18 and 19 years and over were referred to as ‘children’, ‘adolescents’ and ‘adults’, respectively.

Nutrient intakes from each eating event were calculated as follows: the weight of food/beverage consumed in each eating event multiplied by micronutrient concentration in that food/beverage. They were then summed up per person, per day and per micronutrient. Micronutrient intakes were calculated for each participant's base diet only (only non-fortified foods), base diet plus fortified foods (no supplements) and the base diet, fortified foods and dietary supplements together (total foods). These values were divided by the number of consumption days to get an average daily micronutrient intake from each food category over the 4-day survey period per person.

### Statistical analyses

The percentage not meeting the UK DRVs^(13)^ was calculated using the cut-point method by comparing micronutrient intakes with DRVs, i.e. the Estimated Average Requirements (EARs), Lower Reference Nutrient Intake (LRNIs) and Upper Levels of Intake (ULs)^(14)^. This percentage was calculated for each age and gender group and for the base diet only, the base diet plus fortified foods, and the base diet, fortified foods and supplements. When no EAR was available, the percentage below the LRNI was calculated instead (iodine, potassium and selenium), representing those who will almost certainly be deficient^(13)^. Since no EAR or LRNI has been established for vitamin D in the UK only the proportion below the RNI was calculated for this vitamin. Since no DRVs for biotin, vitamin E, vitamin K and pantothenic acid have been set in the UK, these vitamins were not evaluated. The UK DRVs for thiamine and niacin are expressed in mg per 1000 kcal and converted to mg per day using the requirements for energy for different age groups in the UK^(13)^. The percentage of the population exceeding the UL was calculated using the UL values for micronutrients as established by the European Food Safety Authority (EFSA)^(15)^. Age and gender categories were chosen to match the UK's DRVs. Mean, variance and frequency calculations included the NDNS weighting variable to correct for differences in sample selection and response^(2)^. Analyses were conducted with SPSS version 26 (IBM, Armonk, NY, USA), using the weighting variable provided in the dataset to produce nationally representative estimates.

## Results

Table 1 shows the participant number and percentage of total per age group, and the proportion of the 2546 UK participants consuming a base diet only, fortified foods (with or without supplements) and food supplements (with or without the base diet and fortified foods). Overall, 26 % of participants consumed a base diet only without taking a fortified foods or supplements, 72 % of the participants consumed fortified foods and 23 % took a food supplement containing vitamins and/or minerals. Fortified food consumption was highest among children and adolescents aged 1–14 years, while food supplement use was highest among adults, particularly in the 51+ year age category. Supplementary Table S1 shows the number and proportion of fortified food consumers and the type of fortified foods consumed per fortified micronutrient.

**Table 1.** Number and percentage of survey population by age group, and percentage consuming fortified foods and dietary supplements

Age and gender category	Number^a^	% of survey population^a^	% Consuming base diet only^b^	% Consuming fortified foods ± supplements^b^	% Consuming supplements ± fortified foods^b^	% Consuming base diet ± fortified foods ± supplements^b^
1–3 years	215	3·2 %	2·1 %	97·9 %	24·3 %	100 %
4–6 years	245	3·8 %	3·7 %	96·3 %	20·9 %	100 %
7–10 years	250	4·4 %	6·8 %	92·1 %	17·0 %	100 %
11–14 years, male	134	2·4 %	12·1 %	87·9 %	9·2 %	100 %
11–14 years, female	133	2·5 %	17·9 %	77·7 %	9·6 %	100 %
14–18 years, male	134	22·2 %	15·6 %	83·8 %	5·6 %	100 %
14–18 years, female	147	16·2 %	20·3 %	78·6 %	17·1 %	100 %
19–50 years, male	249	2·2 %	31·8 %	67·0 %	17·3 %	100 %
19–50 years, female	433	2·2 %	27·3 %	68·9 %	23·5 %	100 %
51+ years, male	254	21·6 %	31·0 %	63·6 %	26·6 %	100 %
51+ years, female	352	19·1 %	29·6 %	67·3 %	33·3 %	99·2 %
Total	2546	100 %	25·7 %	71·6 %	22·8 %	99·9 %

a Unweighted.

b Weighted.

### Mean intakes in the total population

Table 2 shows the mean intakes of energy, vitamins and minerals consuming a base diet only, a base diet plus fortified foods (but no supplements) and fortified foods plus supplements, in the entire survey population. Fortified foods represented 4 % of the total energy of the UK diet. Fortified foods provided 0–13 % of the total mean intakes for minerals, and 3–15 % of the total mean intakes for vitamins. Of all micronutrients, foods fortified with folic acid contributed most to the mean total intakes. Mineral-containing supplements supplied 0–7 % of total mineral intakes and vitamin-containing supplements supplied 5–40 % of total vitamin intakes (Table 2). Of all micronutrients, vitamin B12-containing supplements contributed most to the total intakes of this vitamin.

**Table 2.** Mean intake of energy, vitamins and minerals in the UK population from the base diet, fortified foods and food supplements^a^

	Total intake		Base diet		Fortified foods		Supplements		Fortified foods	Supplements
	Mean	sd	Mean	sd	Mean	sd	Mean	sd	% of total	% of total
Energy (kJ/d)	7369	2297	7073	2275	292	339	4·9	19·4	4 %	0·1 %
Energy (kcal/d)	1761	549	1690	544	70	81	1·2	4·6	4 %	0·1 %
Calcium (mg/d)	834	345	780	311	32	70	22	136	4 %	3 %
Copper (mg/d)	1·15	0·69	1·08	0·67	0·04	0·06	0·03	0·17	3 %	3 %
Iodine (mg/d)	0·16	0·11	0·15	0·08	0	0·01	0·01	0·08	0 %	6 %
Iron (mg/d)	10·5	5·1	8·5	3·3	1·4	1·9	0·6	3·4	13 %	6 %
Magnesium (mg/d)	251	96	235·5	88·9	10·6	15·9	4·9	28·1	4 %	2 %
Potassium (mg/d)	2673	876	2612	871	61	88	0·4	4·4	2 %	0 %
Selenium (mg/d)	48	26	45	21	0·9	1·5	2·2	15·1	2 %	5 %
Zinc (mg/d)	8·6	4·2	7·8	2·9	0·2	0·5	0·6	2·9	2 %	7 %
Vitamin A (RAE, mg/d)	0·95	1·10	0·85	1·05	0·03	0·08	0·08	0·26	3 %	8 %
Thiamine (mg/d)	2·0	4·0	1·3	0·5	0·2	0·2	0·5	3·9	8 %	27 %
Riboflavin (mg/d)	2·0	2·9	1·4	0·6	0·2	0·3	0·4	2·7	10 %	19 %
Niacin (NE, mg/d)	35	14·8	30·6	11·7	2·7	4·7	1·7	6·5	8 %	5 %
Pantothenic acid (mg/d)	6·7	7·7	5·2	2·1	0·4	1·0	1·1	6·9	7 %	16 %
Vitamin B6 (mg/d)	2·4	4·1	1·6	0·8	0·3	0·6	0·5	4·0	12 %	23 %
Folate (DFE, mg/d)	0·26	0·28	0·19	0·08	0·04	0·05	0·03	0·26	15 %	12 %
Vitamin B12 (μg/d)	8·8	62·4	4·9	3·7	0·4	0·9	3·5	61·9	4 %	40 %
Vitamin C (mg/d)	99	119	75	48	3	11	21	105	3 %	21 %
Vitamin D (μg/d)	4·3	9·6	2·3	2·0	0·4	0·8	1·6	9·3	10 %	36 %
Vitamin E (mg/d)	11·4	18·4	8·4	3·5	0·6	1·3	2·4	17·8	5 %	21 %

RAE, Retinol Activity Equivalents; NE, Niacin Equivalents; DFE, Dietary Folate Equivalents.

a NDNS survey years 5 and 6, *N* = 2546 participants.

### Proportion of the total population above the UL

Less than 1 % of the total UK population exceeded the UL from all sources (base diet, fortified foods and supplements) for any of the vitamins and minerals (data not shown). Across the age categories, children aged 1–3 years most frequently exceeded the UL from the base diet only; copper: 2·8 %, folate: 5·3 %, iodine: 15·6 % and zinc: 4·6 % and from both base diet and fortified foods; copper: 4·4 %, folate: 17·2 %, iodine: 16·6 % and zinc: 10·6 %. Of the children aged 4–6 years, 2·1 and 6·0 % exceeded the UL for folate from the base diet and fortified foods and additional supplements, respectively.

### Proportion of the total population below the EAR or LRNI for minerals and vitamins

**Fig. 1.**
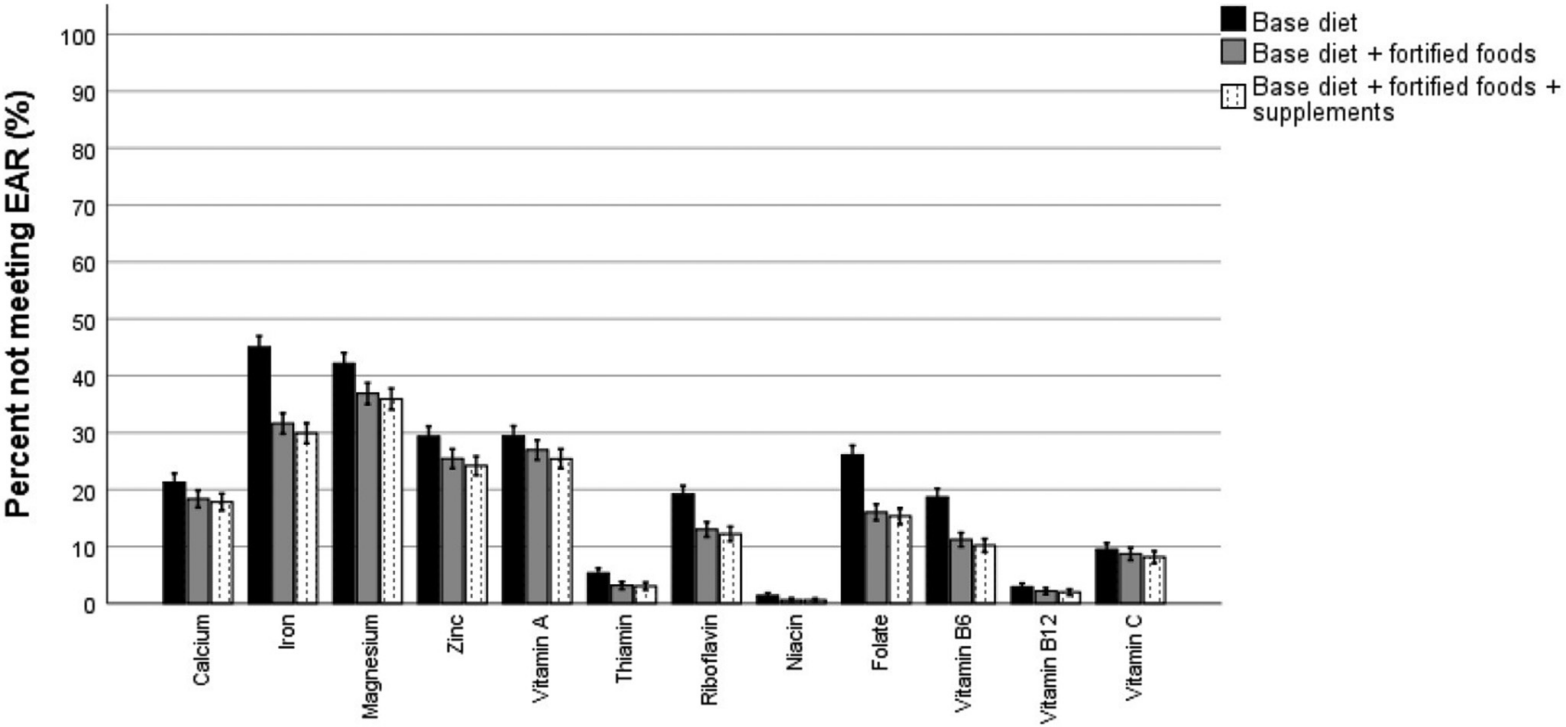
Proportion of the UK population not meeting the EAR for micronutrients.

**Fig. 2.**
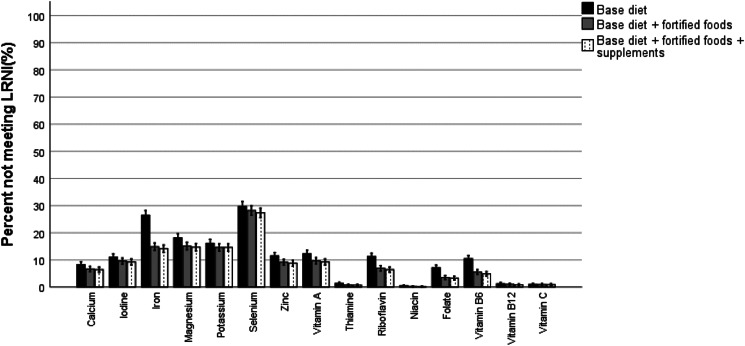
Proportion of the UK population not meeting the LRNI for micronutrients.

The percentage of the total population consuming a base diet only that failed to meet the EAR was 21 % for calcium, 45 % for iron, 42 % for magnesium and 29 % for zinc (Fig. 1). The proportion of the population consuming a base diet only that failed to meet the EAR was 29 % for vitamin A, between 19 and 26 % for riboflavin, vitamin B6 and folate and <9 % for thiamine, niacin and vitamin B12 and C (Fig. 1). Lower inadequacy rates were observed for those consuming additional fortified foods, while supplements contributed marginally to reducing inadequacy. Particularly fortification with iron, magnesium, zinc, vitamin A and B vitamins helped to reduce the prevalence of inadequacy. Mineral intakes fell below the LRNI for 7–30 % of the population consuming a base diet only and were highest for selenium (30 %) (Fig. 2). Fortification, but not supplements, reduced the percentage with mineral intakes below the LRNI with a few percent. This was most notably for iron that reduced inadequacy prevalence from 27 % in those on a base diet to 15 % in those consuming additional fortified foods. The proportion of participants on a base diet alone with vitamins intakes below the LRNI ranged from 0 to 11 %. The proportion of the population consuming fortified foods or supplements who were inadequate decreased by a few percentage points (Fig. 2).

### Proportion of the population subgroups below the EAR or LRNI for macrominerals

About 4–9 % of young children aged 1–10 years consuming a base diet only fell short of their calcium requirements (Supplementary Fig. S1). This was reduced to 2–5 % in those consuming additional fortified foods. Calcium intakes were below the EAR for almost 60 % of women aged 11–18 years only consuming a base diet (Supplementary Fig. S1). Foods fortified with calcium reduced the proportion below the EAR by 10 %. Calcium-containing supplements barely contributed to meeting the requirements. Iodine intake below the LRNI was also most prevalent among age 11–18 years, ranging from 15–19 % in young men to 24–30 % in young women from the base diet (Supplementary Fig. S2). Foods or supplements with added iodine barely reduced the percentage below the LRNI. Particularly, women and men aged 11–18 years were largely below the EAR for magnesium when considering the base diet (Supplementary Fig. S3); 60 % to almost 90 % of the men and women aged 11–18 years had magnesium intakes below the EAR from the base diet. Magnesium-fortified foods (but not supplements) helped to reduce the proportion below the EAR by several percent. In participants from the base diet only, the LRNI for potassium was not achieved by 23–40 % of women and 9–23 % in men aged 11 years and older (Supplementary Fig. S4). Fortified foods and food supplements reduced the percentage falling below the potassium LRNI by a few percent.

### Proportion of the population subgroups below the EAR or LRNI for trace minerals

A large proportion of children 1–3 years (82 %), women 11–50 years (33–98 %) and men 11–18 years (51–76 %) had iron intakes below the EAR from the base diet (Supplementary Fig. S5). Foods fortified with iron, but not supplements containing iron, reduced the proportion of adolescent women not meeting the requirements considerably by almost 20 %. About 48–71 % of women aged 15 and older and 25–31 % of men aged 11 years and older failed to meet the LRNI for selenium from the base diet (Supplementary Fig. S6). In the population consuming fortified foods (but not supplements), selenium intakes below the LRNI were several percentages lower. Approximately 54–64 % of men and women aged 11–14 years and 24–36 % of older ages had zinc intakes below the EAR from the base diet (Supplementary Fig. S7). Fortified foods but not supplements with zinc considerably helped in meeting the EAR, particularly children aged 1–10 years.

### Proportion of the population subgroups below the EAR or LRNI for vitamins

The prevalence of vitamin A intakes below the EAR ranged between 16 and 29 % for ages <6 years and ranged between 19 and 47 % for older ages consuming a base diet alone (Supplementary Fig. S8). Fortified foods and food supplements reduced inadequate vitamin A intakes by a few percent.

Less than 13 % of all participants not consuming any fortified foods or supplements had intakes below the EAR for thiamine, niacin and vitamin B12 (not shown). Inadequacies were more frequent for riboflavin, vitamin B6 and folate. The proportion of children with inadequate intakes of riboflavin from the base diet was in 21 % of children aged 7–10 years, which was reduced to 7 % if considering intakes from fortified foods as well. The EAR for riboflavin was not met by 10–45 % of the population aged 11 years and older from the base diet (Supplementary Fig. S9). Riboflavin-fortified foods significantly reduced the proportion of inadequate individuals 11 years and older. The vitamin B6 intake requirements were not reached by 12–41 % of those aged 11 years and older consuming a base diet only (Supplementary Fig. S10). Fortified foods decreased inadequacies in this group to 7–24 % while supplements contributed little extra. The proportion of the population with folate intakes below the EAR was higher in women than men and declined with age; 59–65 % of adolescent boys and girls aged 11–18 years relying on a base diet failed to meet their requirement (Supplementary Fig. S11). The percentage of girls and boys aged 11–18 years below the EAR was at least 20 % lower in those taking fortified foods, while supplements did not make a meaningful contribution to intakes.

Most children below age 11 achieved their EAR for vitamin C, while 6–12 % of all individuals aged 11 years and older had vitamin C intakes below the EAR (Supplementary Fig. S12). Neither fortified foods, nor supplements with vitamin C significantly reduced the percentage of individuals below the EAR.

More than 99 % of the UK population failed to meet the RNI for vitamin D from the base diet (Supplementary Fig. S13). Fortified foods did not significantly decrease the proportion of people with vitamin D intakes below the RNI. Vitamin D inadequacy was 94 % in supplement users and 87 % in supplement users over the age of 50.

## Discussion

This is the first in-depth analysis to our knowledge that differentiates between mineral and vitamin intakes in the UK from the diet alone, voluntarily fortified foods and supplements for different age groups and genders. The present study revealed that most of the UK population aged 11 years and older, particularly women aged 11–18 years, have inadequate intakes from all dietary sources for all minerals analysed (calcium, iodine, iron, magnesium, potassium, selenium and zinc), and most of the vitamins analysed (vitamin A, riboflavin, vitamin B6, folate and vitamin D).

Analyses including earlier UK survey years as of 2008/9 show that adult micronutrient intakes of all minerals, vitamin A, riboflavin and folate were below the LRNI level^(4)^ with even a downward trend in intakes of most vitamins and minerals over the 9 survey years^(16)^. In accordance with our findings, the results of the UK national report of the same survey years 2012/13–2013/14 showed low intakes of all minerals, and vitamin A and riboflavin by adolescents (mostly girls) in the UK^(2)^. These data suggest that mineral and most vitamin intakes are generally inadequate in the UK.

Of all packaged food and drink products in the UK market (except for baby foods and meal replacers), about 20 % were fortified with at least one vitamin or mineral during the survey years^(17)^. Our analysis of UK's survey shows that over 70 % of individuals in the UK consume voluntarily fortified foods. More participants consuming a base diet only failed to meet the micronutrient requirements as compared to those consuming additional fortified foods. In particular, a large proportion of children aged 1–10 years achieved their micronutrient requirements from voluntarily fortified foods such as cereals and juices. In ages 11 years and older, fortified foods mainly helped to address iron, riboflavin, vitamin B6 and folate inadequacies. In Ireland, fortified foods similarly made a considerable contribution to intakes of vitamins in young children^(18)^ and older adults^(19)^.

In contrast to fortified foods, supplements only marginally reduced the prevalence of inadequate intakes across age groups. The proportion of participants taking a vitamin/mineral supplement during the past 4 d in this and earlier surveys^(20)^ is rather low (23 %) and may not reduce inadequacies. Therefore, fortified foods may be more likely than supplements to reach those individuals at risk of micronutrient inadequacies. Food supplement use tends to be higher among those with healthier lifestyles and may be consumed little by to those individuals with inadequate dietary intakes^(21,22)^.

Only few foods naturally contain vitamin D, as confirmed by this analysis showing that 99 % of the UK population failed to meet the reference intakes from the diet alone. Vitamin D-fortified foods did not contribute to reducing inadequacies since also 99 % of fortified food consumers failed to meet their reference intakes. These vitamin D inadequacy rates are comparable with those observed in other European countries ranging from 97 to 100 %^(23,24)^. In most European countries, only 1–2 % of products in the market are fortified with vitamin D^(17)^ and they may barely contribute to reducing inadequacy^(24)^. However, in countries with large-scale voluntary vitamin D fortification such as Finland and the US, fortified foods are major contributors to meeting adequate vitamin D intakes^(25)^. A more large-scale approach through universal fortification of commonly consumed foods appears to be a successful strategy to reduce vitamin D deficiency, as has been shown in Finland^(26)^. Supplements only helped 6 % of the population to achieve their recommended intakes for vitamin D, probably because supplement use was relatively low at the time of the survey (23 %). The UK survey of the same years showed that around 20 % of adults were vitamin D deficient (<25 nmol/l)^(2)^, suggesting that dietary sources and sunlight exposure together were insufficient to sustain serum vitamin D levels necessary for musculoskeletal health. Since 2016, however, all people in UK over age 1 year are recommended to take a vitamin D supplement in autumn and winter. Calcium intake from all dietary sources including voluntarily fortified foods and supplements was too low for one fifth of UK adolescents in this analysis and is also likely too low for musculoskeletal health, especially if vitamin D status is limiting. Voluntary fortified dairy and mandatory fortified flour are main sources of calcium in the UK diet^(27)^. The present study accordingly found that foods voluntarily fortified with calcium helped reducing calcium inadequacies, particularly in adolescents.

Over one-third of UK adolescents had vitamin A intakes below their requirements. Fortified foods and supplements barely contributed to reducing the inadequate vitamin A intakes. Margarine consumption is declining, therefore, large-scale fortification of cooking oils with vitamin A could be considered in the UK. In the US, voluntary fortification of milk with vitamin A has mitigated the risks of vitamin A inadequacy^(28)^.

The present study shows that most male and female adolescents failed to meet their iron, vitamin B6 and folate requirements if relying only on a base diet. Data also show that 4 % of women aged 15–49 years in the UK had iron-deficiency anaemia^(29)^. Meat is the main source of haem iron, vitamin B6 and B12. Strategies aimed at encouraging reduced meat consumption for environmental concerns may have negative consequences for the intake of these micronutrients by women. However, the present study shows that voluntary fortification of foods with iron, folate and other B vitamins reduced a meaningful proportion of British people, particularly adolescent women, who had inadequate intakes. Current iron supplement use is probably very low as adolescent and adult women in UK are not recommended iron supplements^(30)^. The present study also shows that folic acid supplements are not lowering inadequacy rates in women of childbearing age despite the advice in the UK that all women who could become pregnant to take a folic acid supplement. Surveys confirm that only around one quarter of pregnant women in the UK take a prenatal folate supplement^(31)^. Strategies for this group to take and adhere to iron and folic acid supplements, or even multi-micronutrient supplements^(32)^, may be considered given the high micronutrient inadequacies and requirements for this group^(33)^.

About 19–30 % of the UK adolescents consume less than the LRNI for iodine, with adolescent women most likely to have low intakes. Studies of iodine status in particular population groups in the UK confirm that iodine status in pregnant women is deficient^(34,35)^. Over 70 % of adolescent men and women are not achieving their requirements for magnesium. Selenium intakes were particularly a concern in adolescent females, 40 % of them having intakes below the LRNI, suggesting that they will almost certainly be deficient. UK cereals are relatively low in selenium^(36)^. Zinc intakes were also particularly low in adolescents, with at least half of them not meeting the requirements. Current voluntary fortification practices in UK do not help reduce iodine, magnesium, selenium and zinc inadequacies to a significant extent. A large proportion of UK's population did not meet their recommended intakes for potassium, in line with global observations^(37)^. Large-scale fortification with iodine, magnesium, selenium and zinc may support intakes of these shortfall nutrients across the entire population. The potential to fortify foods with potassium is limited due to negative sensory attributes and the large amounts required.

Overconsumption of vitamins and minerals is low in the UK population; less than 1 % of the total UK population exceeded the UL from the base diet, fortified foods and supplements for any of the vitamins and minerals. In children aged 1–3 years, a small percentage consumed copper, folate, iodine and zinc in excess predominantly from the base diet while fortified foods contributed little. Similar findings were reported by an analysis of European datasets from 1997 and 2000–2001, reporting that the 95th percentile intakes of children 4–10 years of these minerals from the base diet alone was close to or over the UL while fortified foods contributed relatively little^(11)^. This is not immediately a reason for concern since the UL comprises an uncertainty margin lower than the intake level at which the first adverse effects may occur to account for uncertainty.

This secondary analysis of the UK NDNS has several limitations. Firstly, food intakes were measured using a 4-day dietary record that may be subject to underreporting of food and thus energy intake. The reported average energy intake of 2297 kcal/d in this survey is nevertheless in line with the expected average energy requirements of 2243 kcal/d for adults with moderate activity level, suggesting no important underreporting. Secondly, the process of recording the diet can influence individuals to change their usual eating patterns towards a healthier diet. Thirdly, while the sample size was large enough to provide a robust estimate of population intakes, the number of children and adolescents was relatively low. Therefore, results should be interpreted with caution for the younger age groups. Lastly, we used a mean of four dietary recall days for which the within-person variation of nutrient intakes is large and may lead to a biased estimation of the fraction of the population above or below a cut-point when it occurs in the tails of the distribution^(38)^, therefore, estimates for proportion of the population not meeting the DRV or above the UL may be exaggerated.

Efforts should continue to encourage the UK population not only to reduce ‘empty’ calorie foods, but also to select nutrient-rich choices. Voluntary fortification is not always tailored to consumer nutritional deficiencies and depends on consumer demand. Governments could raise awareness and encourage food manufacturers to take a more tailored voluntarily fortify with nutrients of public health concern. Governments and healthcare professionals could be instrumental in raising more awareness around the importance of nutrients of concern amongst risk groups that need them the most. Mandatory fortification provides another low-cost means to increase specific nutrients that are challenging to obtain from the diet alone^(39)^. A straightforward approach would be to expand the existing flour fortification legislature to include other micronutrients of concern. In parallel, access to and consumption of nutrient-rich foods could be encouraged possibly via fiscal mechanism. Future research could model the impact of large-scale or voluntary fortification on micronutrient coverage and public health.

## Conclusion

This secondary analysis of this NDNS survey years 2012/13–2013/14 shows a high prevalence of micronutrient shortfalls in the UK population. For women, especially when adolescent, achieving adequate micronutrient intakes is generally more problematic than for men. Results suggest that fortified foods make a modest contribution to reducing the proportion of UK individuals falling short for micronutrient.

## Supplementary material

For supplementary material accompanying this paper visit https://doi.org/10.1017/jns.2022.47. click here to view supplementary material
