# Supplementary material for: Contribution of base diet, voluntary fortified foods and supplements to micronutrient intakes in the UK
Source: J Nutr Sci. 2022 Jun 23;11:e51. doi: 10.1017/jns.2022.47 (PMC9241063; doi:10.1017/jns.2022.47)
Supplement: Supplementary file 1 [file S2048679022000477sup001.docx]

# SUPPLEMENTAL MATERIALS

## Figure S1: Proportion of the UK population not meeting the EAR for calcium


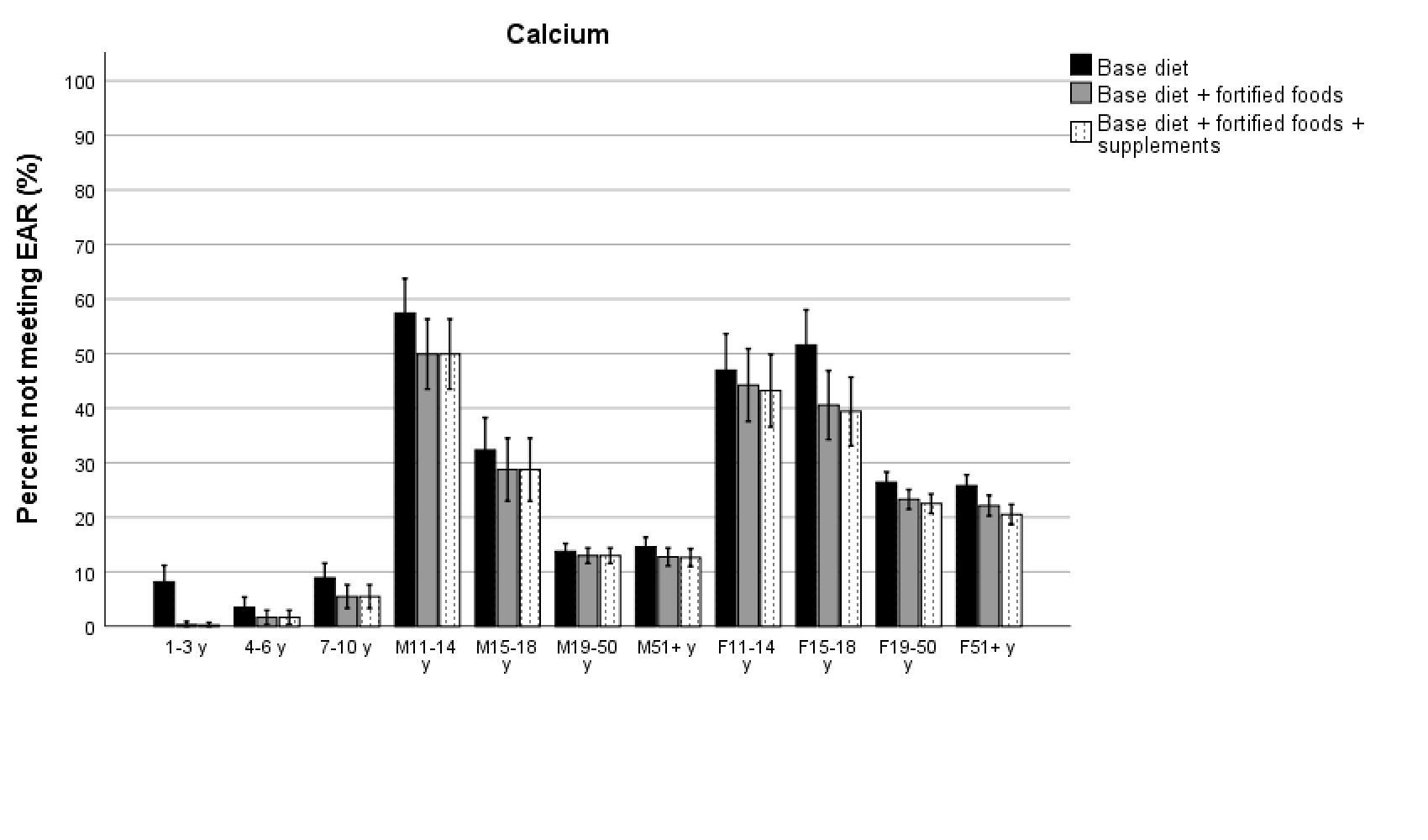


## Figure S2: Proportion of the UK population not meeting the LRNI for iodine


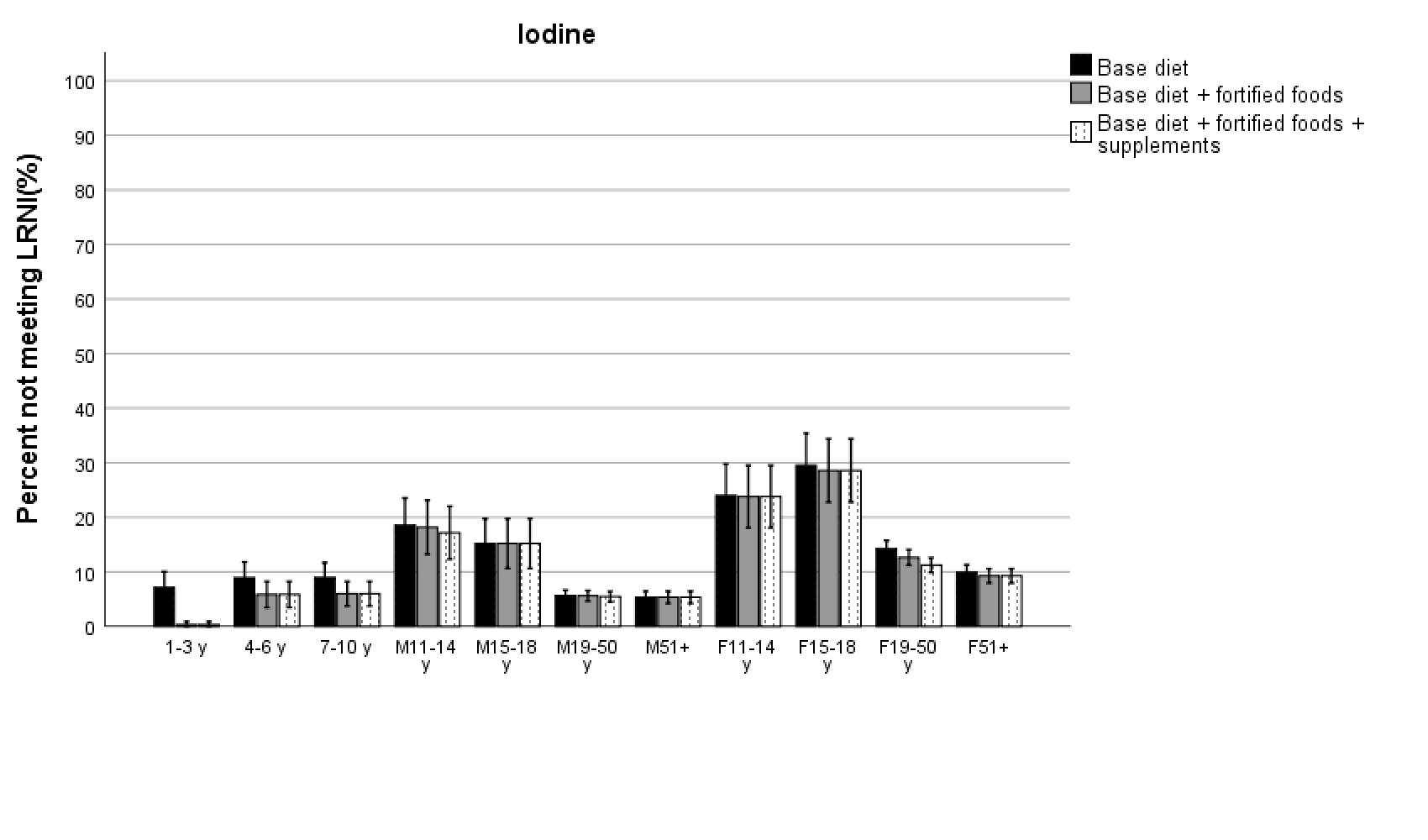


## Figure S3: Proportion of the UK population not meeting the EAR for magnesium


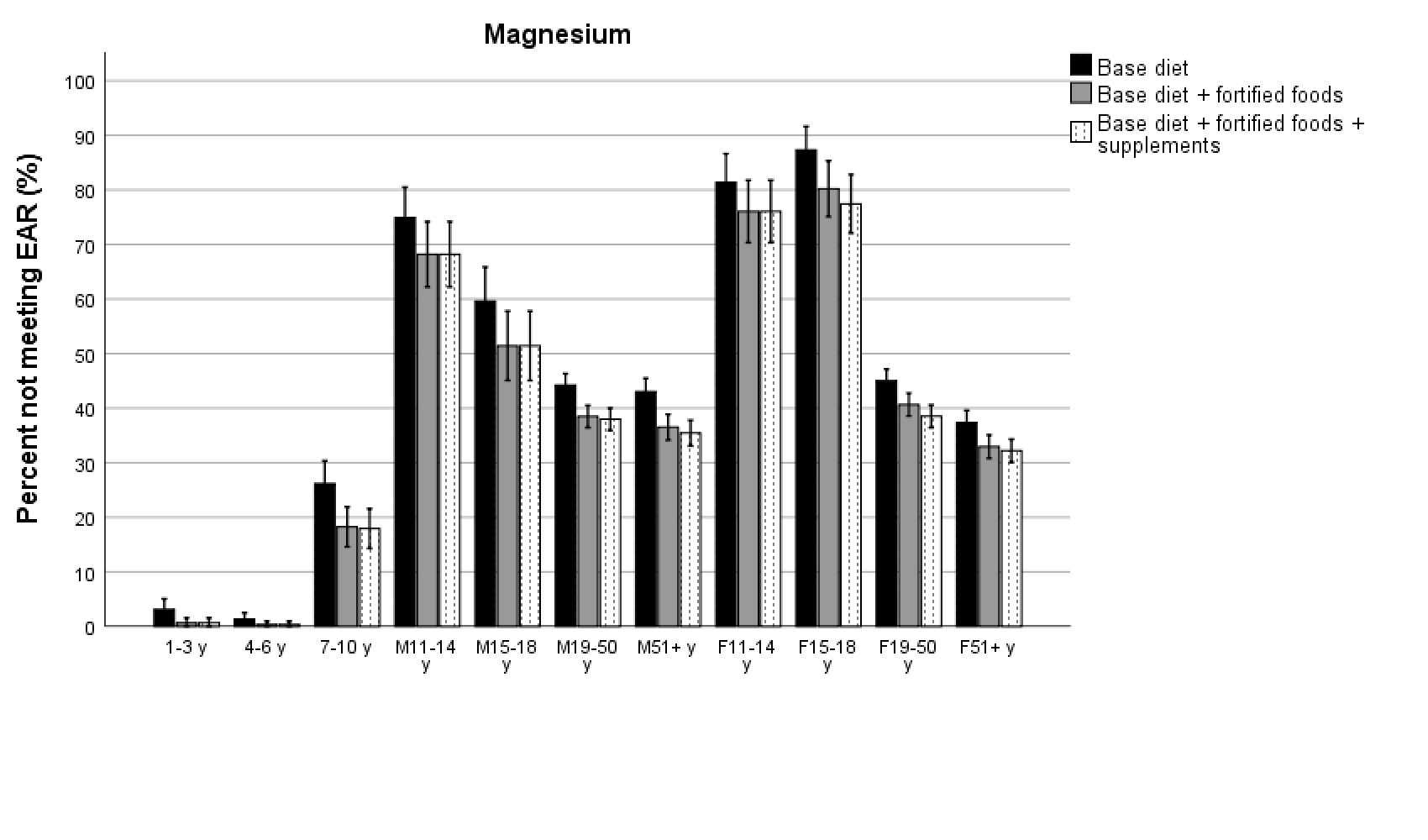


## Figure S4: Proportion of the UK population not meeting the LRNI for potassium


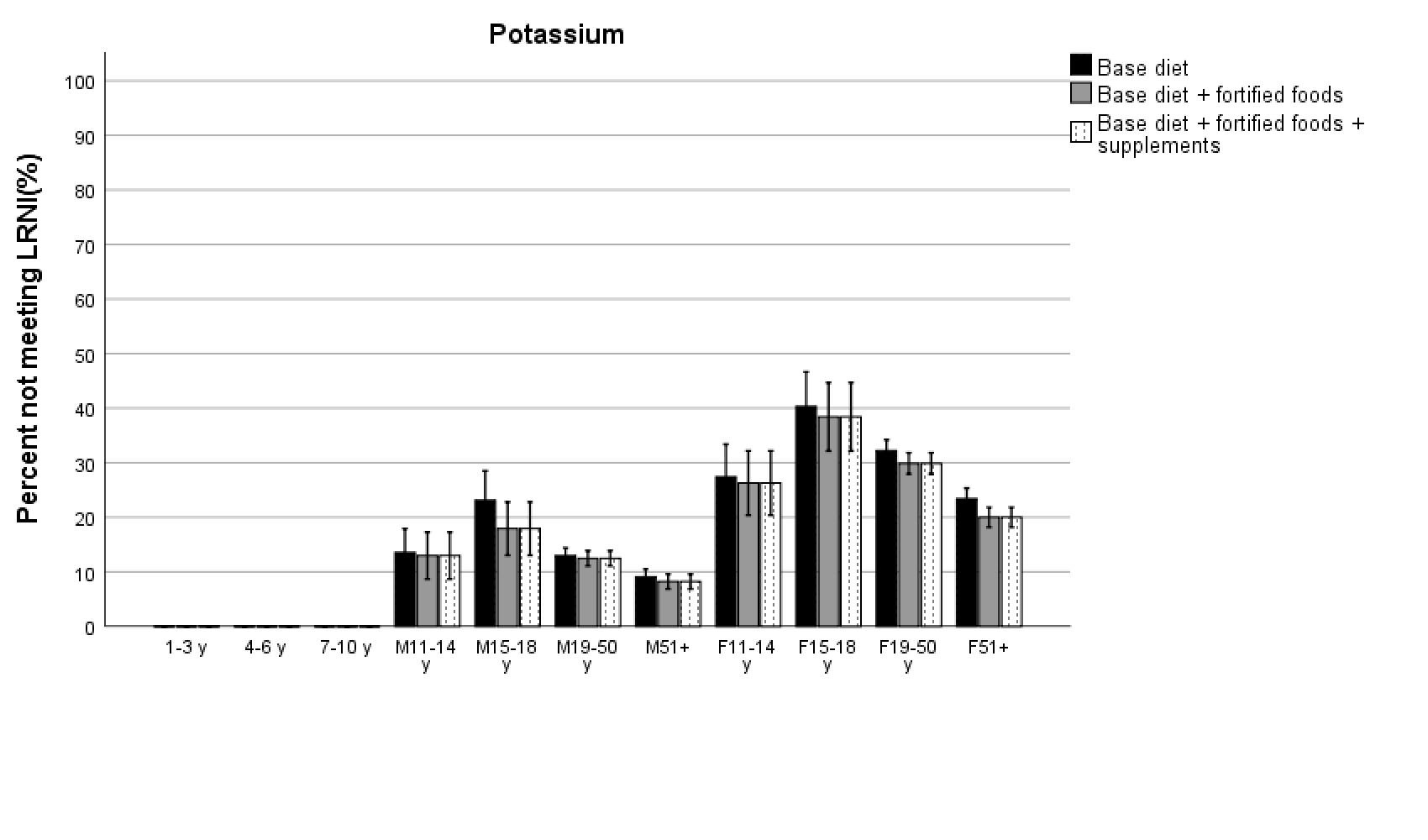


## Figure S5: Proportion of the UK population not meeting the EAR for iron


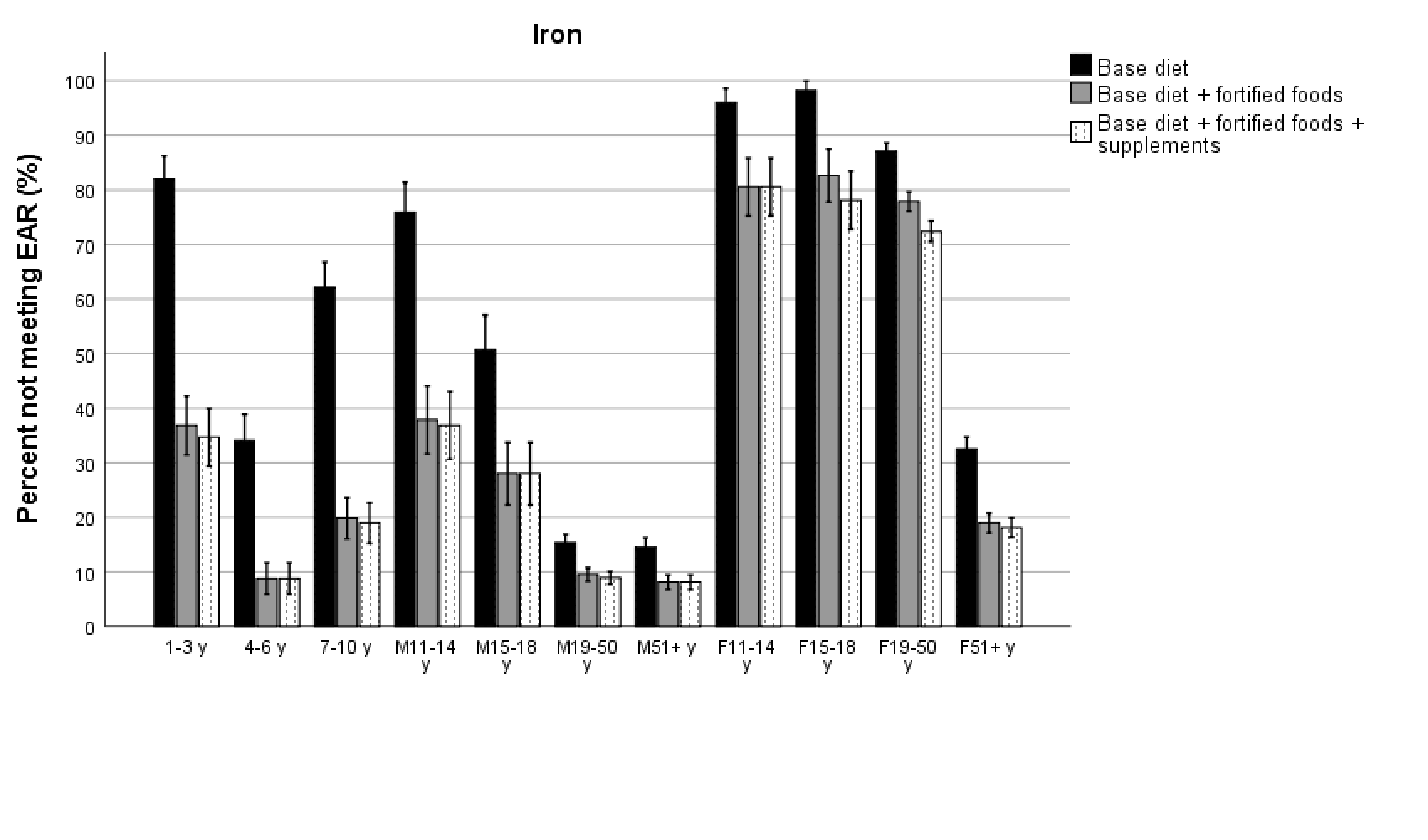


## Figure S6: Proportion of the UK population not meeting the LRNI for selenium


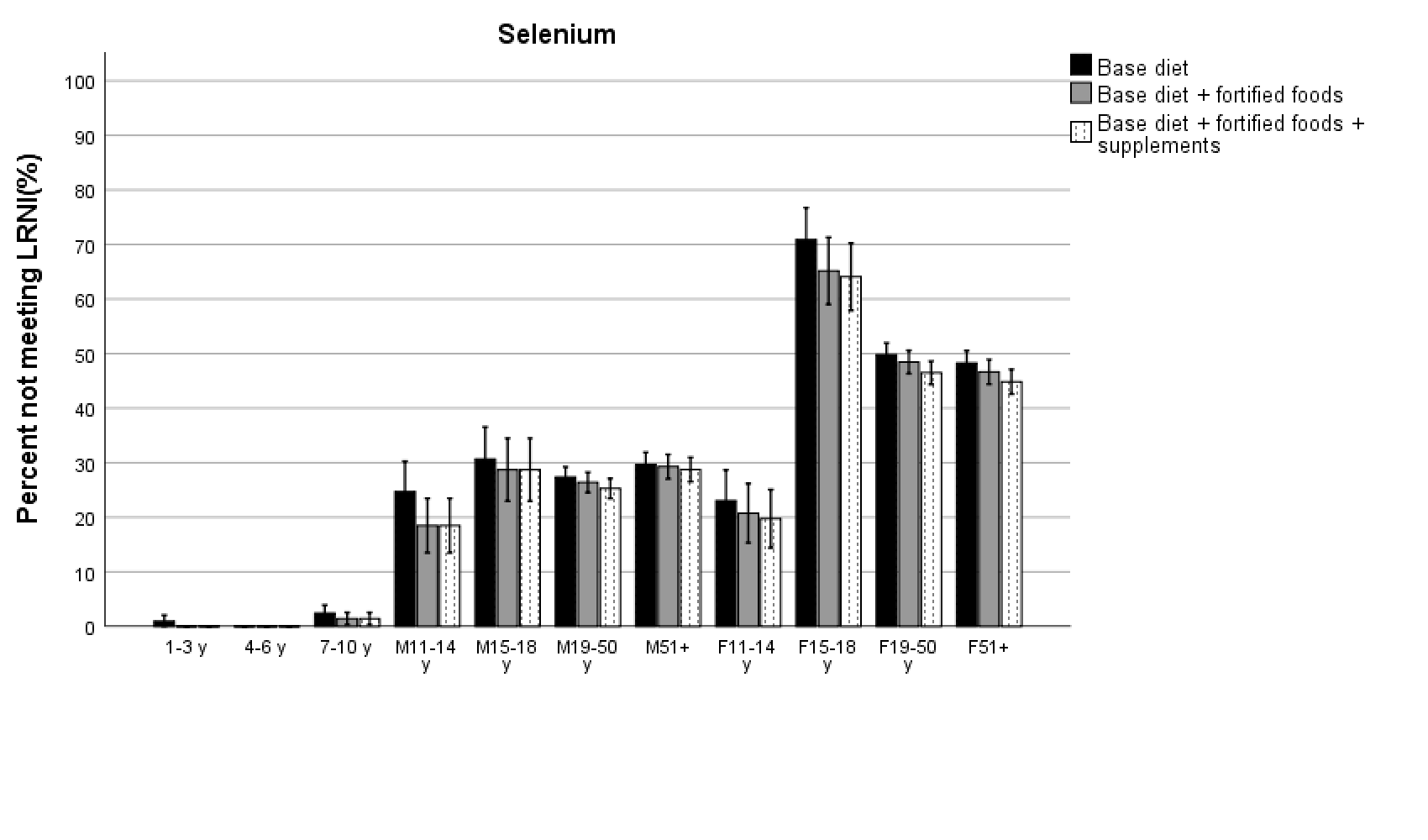


## Figure S7: Proportion of the UK population not meeting the EAR for zinc


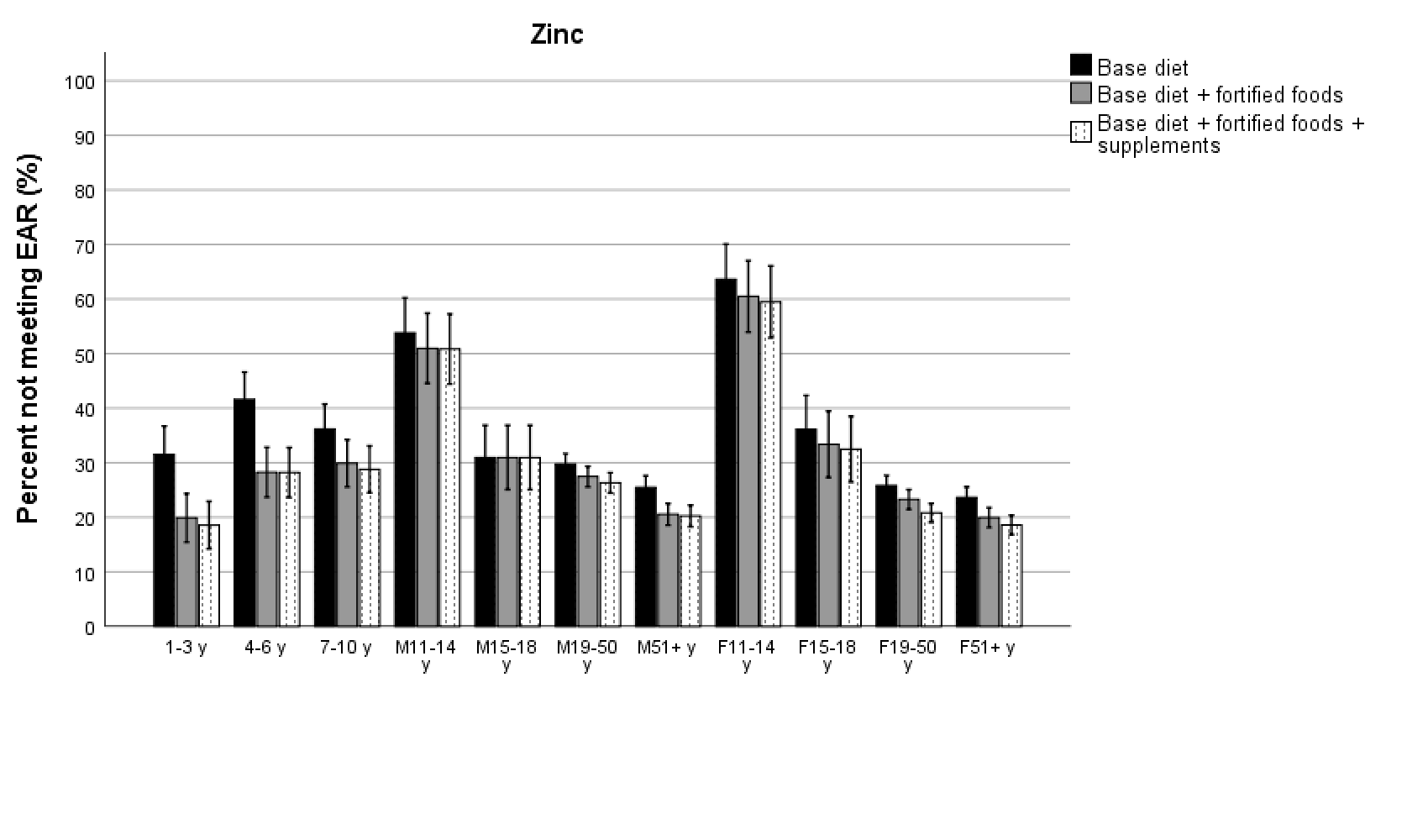


## Figure S8: Proportion of the UK population not meeting the EAR for vitamin A


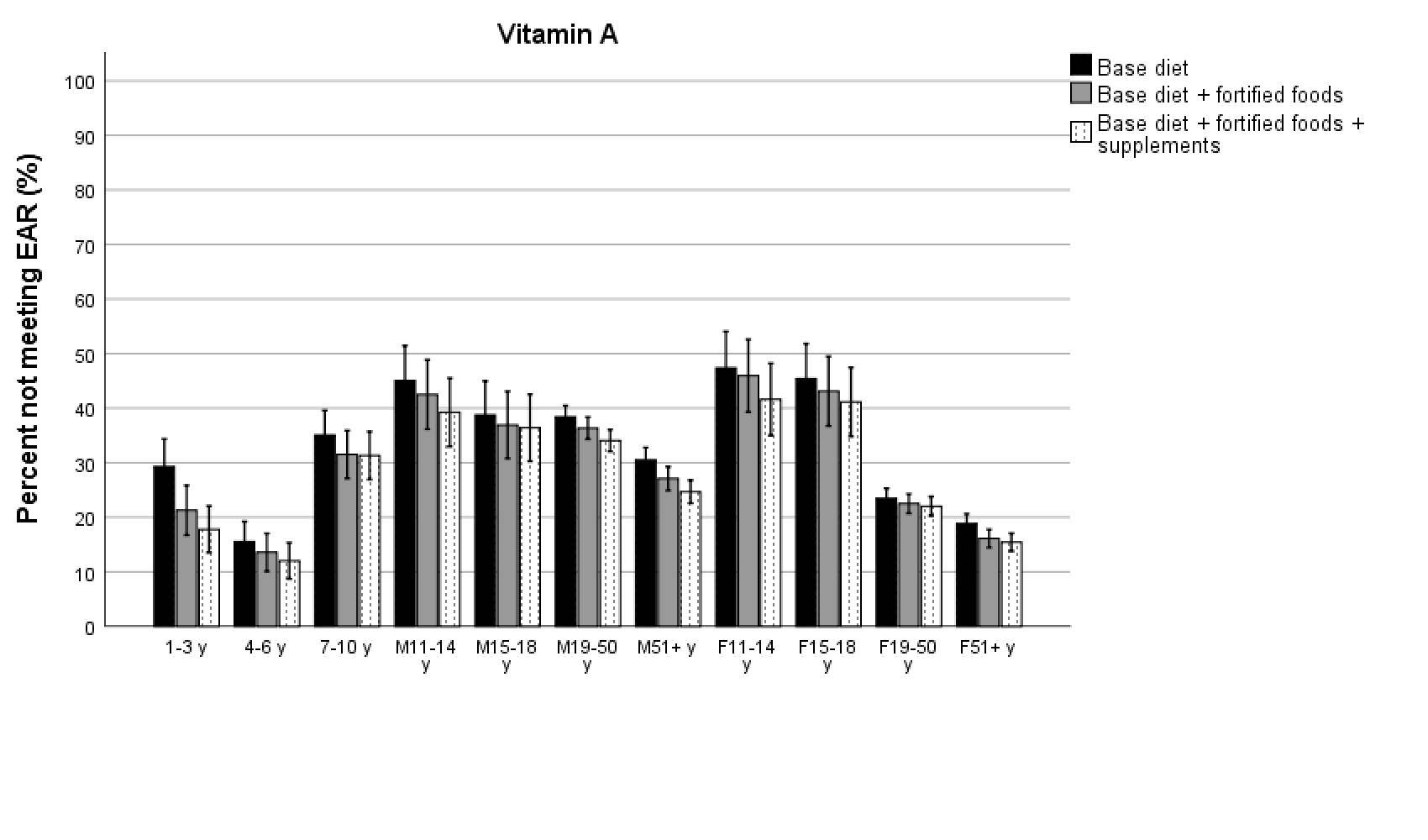


## Figure S9: Proportion of the UK population not meeting the EAR for riboflavin

##
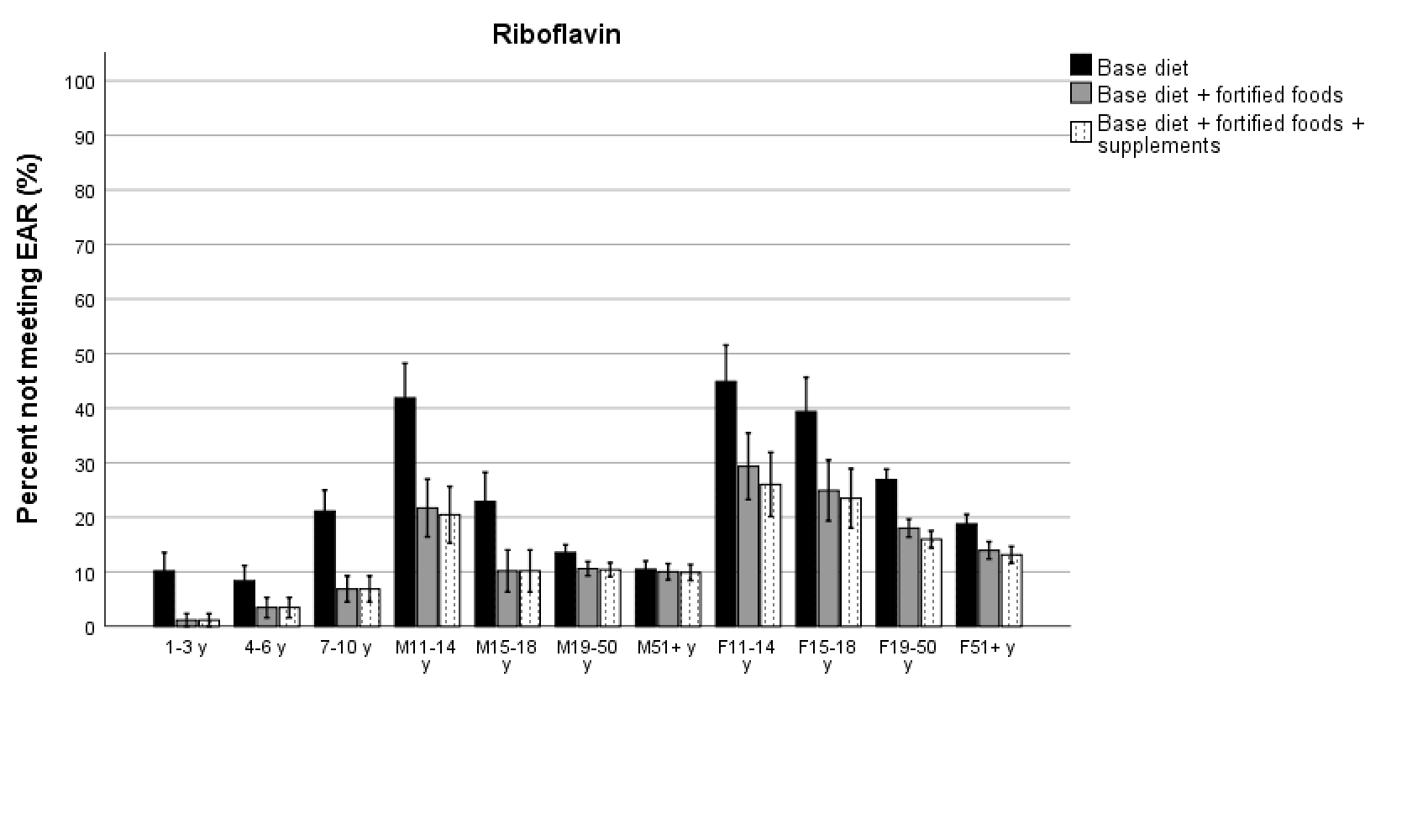


## Figure S10: Proportion of the UK population not meeting the EAR for vitamin B6

##
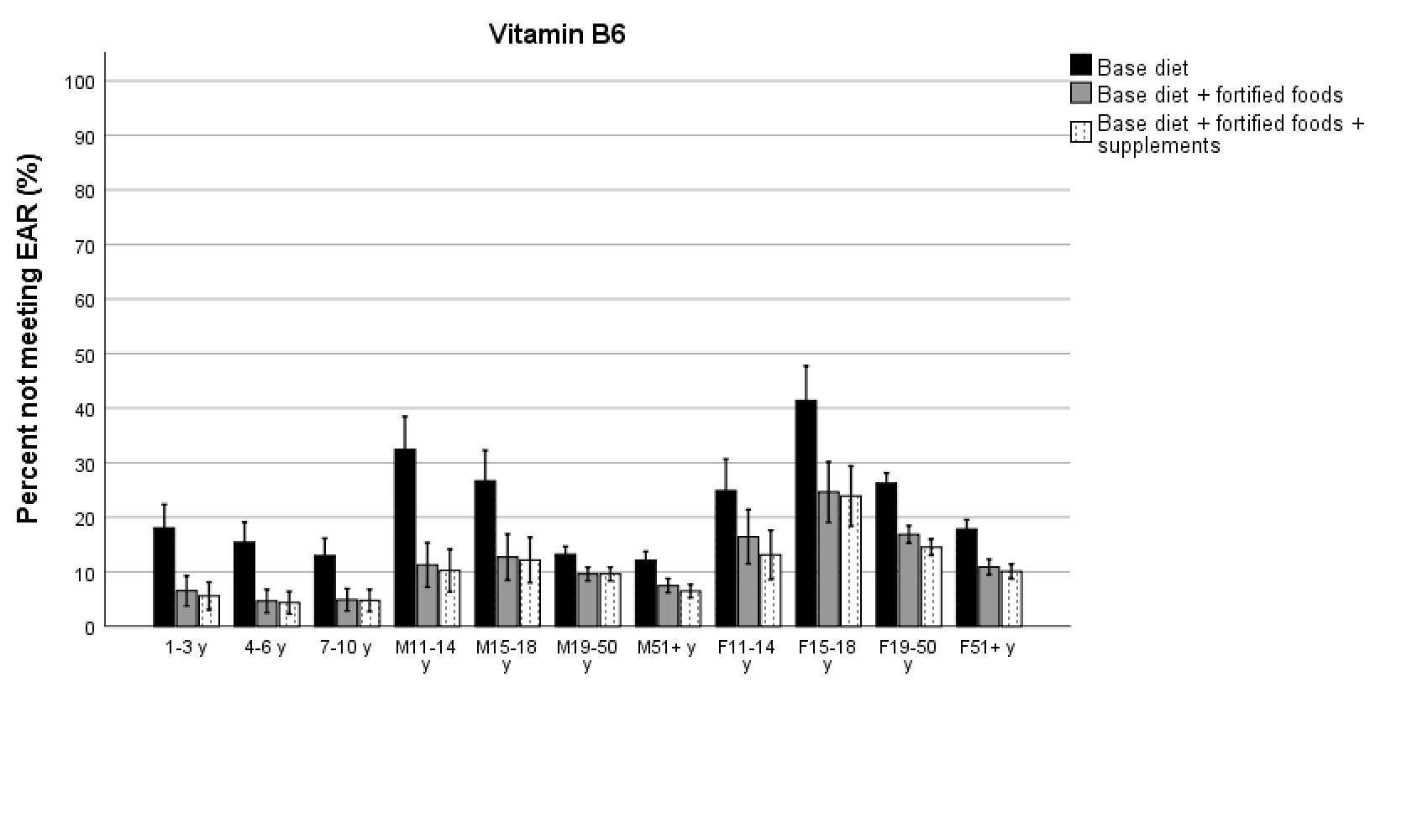


## Figure S11: Proportion of the UK population not meeting the EAR for folate


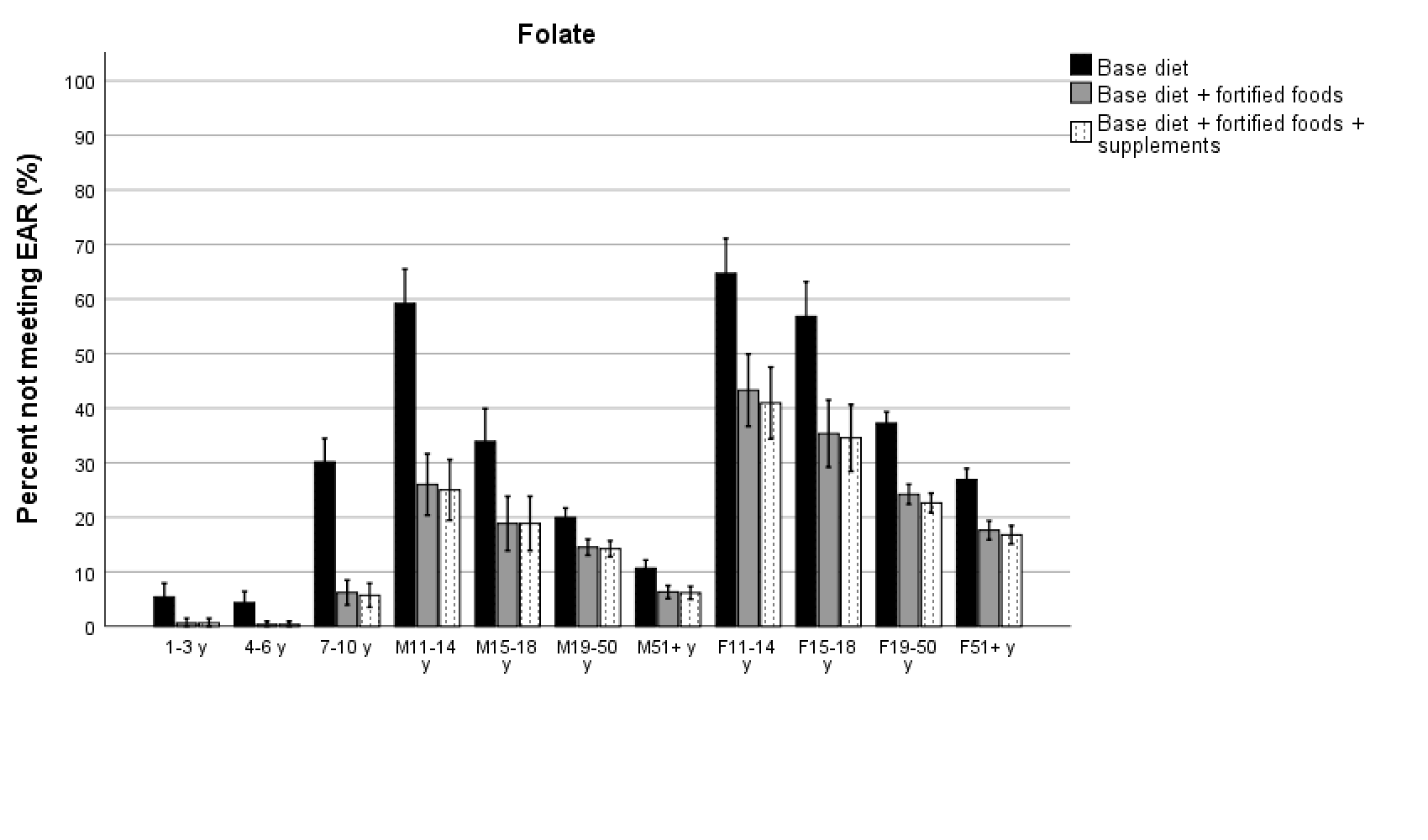


## Figure S12: Proportion of the UK population not meeting the EAR for vitamin C


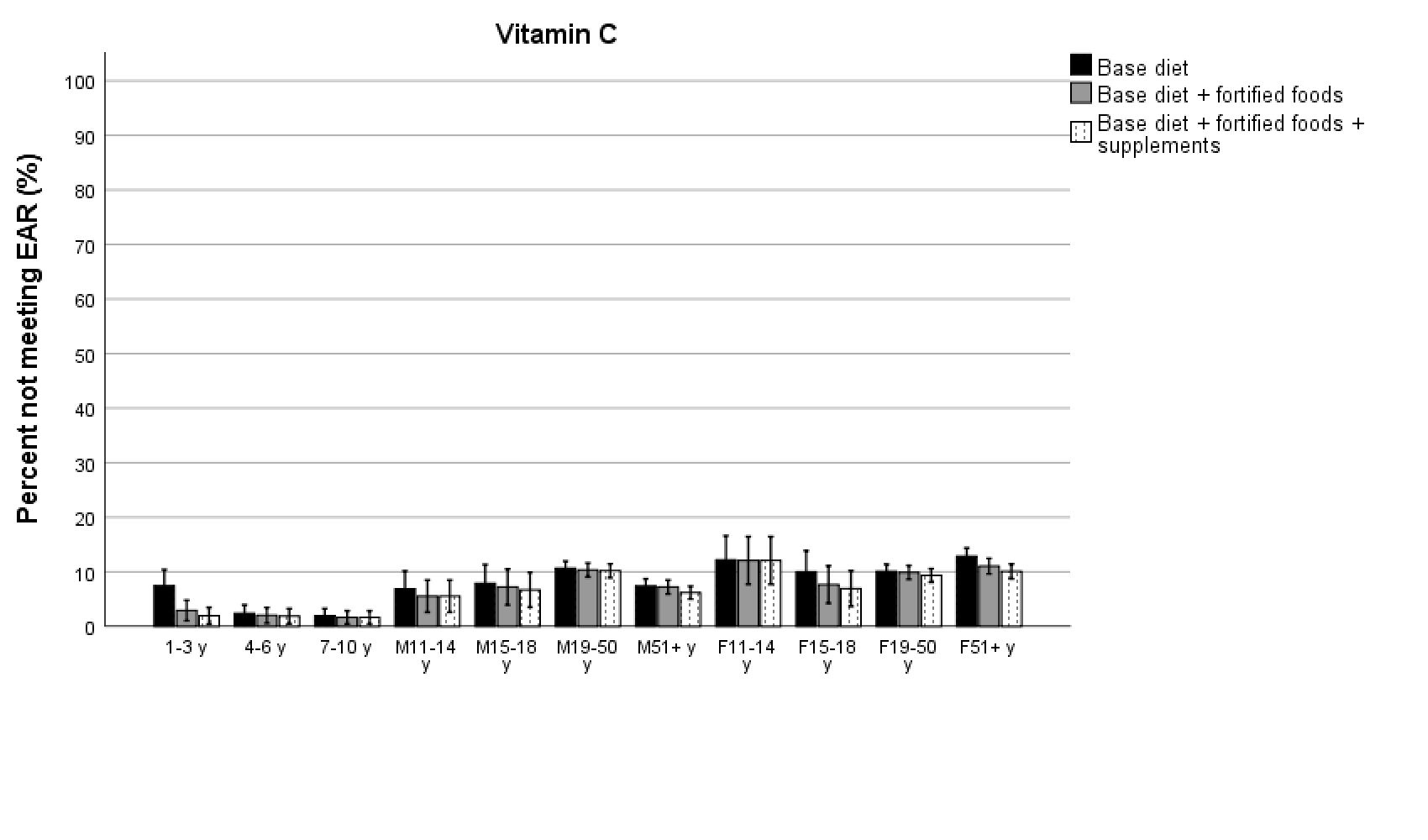


## Figure S13: Proportion of the UK population not meeting the RNI for vitamin D


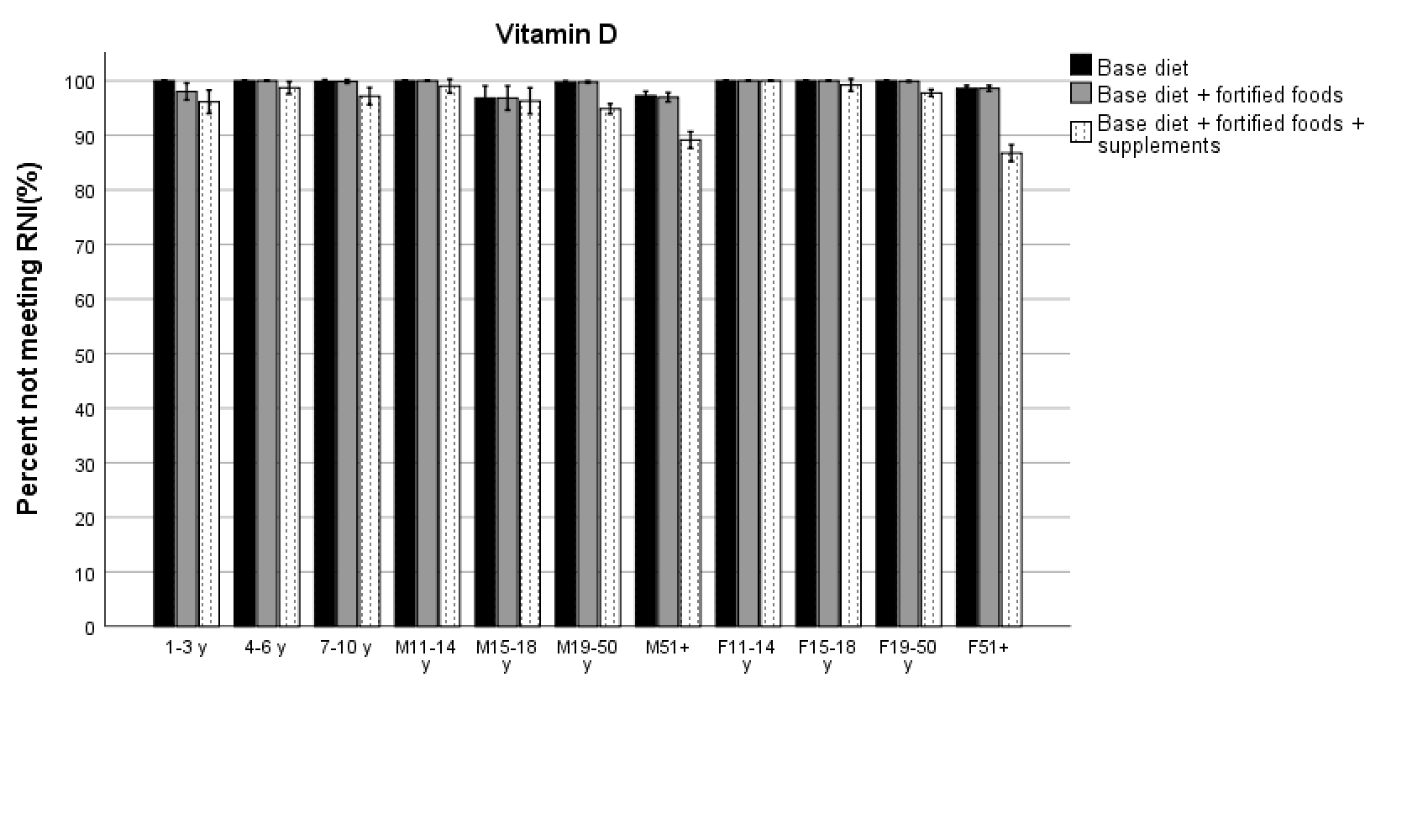


## Table S1: Number and proportion of fortified food consumers and the type of fortified foods consumer per fortified micronutrient

|  | **Undefined** | **Vit A** | **Vit C** | **Vit D** | **Vit B1** | **Vit B2** | **Niacin** | **Vit B5** | **Vit B6** | **Vit B12** | **Vit E** | **Folic acid** | **Calcium** |
| --- | --- | --- | --- | --- | --- | --- | --- | --- | --- | --- | --- | --- | --- |
| Number (proportion) of fortified food consumers | 142  (4.2%) | 531 (21.5%) | 332 (9.7%) | 1,651 (57.7%) | 1,571 (54.7%) | 1,645 (57.3%) | 1,709 (59.6%) | 554 (18.8%) | 1,538 (54.5%) | 1,506 (55.9%) | 75  (3.4%) | 1,713 (61.0%) | 377 (11.9%) |
| Biscuits | X |  |  | X | X | X | X |  | X | X |  | X | X |
| Brown, granary and wheat-germ bread |  |  |  |  |  |  |  |  |  |  |  | X |  |
| Buns, cakes pastries & fruit pies |  |  |  | X |  |  |  |  |  |  |  | X |  |
| Cheese |  |  |  | X |  |  |  |  |  |  |  |  | X |
| Chocolate confectionery |  |  |  |  | X |  |  | X | X | X |  |  | X |
| Commercial toddlers foods and drinks | X | X | X | X | X | X | X | X | X |  | X |  | X |
| Fruit juice | X |  |  |  |  |  |  |  |  |  |  |  | X |
| High fibre breakfast cereals | X |  | X | X | X | X | X | X | X | X |  | X | X |
| Low fat spread |  | X |  | X |  |  |  |  | X | X |  | X |  |
| Miscellaneous |  | X |  | X | X |  | X | X | X | X | X | X | X |
| Other breakfast cereals |  |  |  | X | X | X | X | X | X | X |  | X | X |
| Other milk and cream |  |  |  | X |  | X |  |  |  | X | X |  | X |
| Pasta, rice and other cereals | X |  |  |  |  |  |  |  |  |  |  |  |  |
| Reduced fat spread | X | X |  | X |  | X | X |  | X | X |  | X |  |
